# Supplementary material for: Multi‐dataset identification of innovative feature genes and molecular mechanisms in keratoconus
Source: J Cell Mol Med. 2024 Sep 19;28(18):e70079. doi: 10.1111/jcmm.70079 (PMC11412914; doi:10.1111/jcmm.70079)
Supplement: Supplementary file 2 — Material S2. [file JCMM-28-e70079-s002.zip › 7.GSEA analysis/5.RNF217-AS1/KEGG_PHOSPHATIDYLINOSITOL_SIGNALING_SYSTEM.html]

Details for gene set KEGG\_PHOSPHATIDYLINOSITOL\_SIGNALING\_SYSTEM[GSEA]

|  || Dataset | RNF217-AS1.group.cls#h\_versus\_l |
| Phenotype | group.cls#h\_versus\_l |
| Upregulated in class | l |
| GeneSet | KEGG\_PHOSPHATIDYLINOSITOL\_SIGNALING\_SYSTEM |
| Enrichment Score (ES) | -0.31652072 |
| Normalized Enrichment Score (NES) | -1.3424442 |
| Nominal p-value | 0.13319239 |
| FDR q-value | 0.37190294 |
| FWER p-Value | 0.924 |
Table: GSEA Results Summary

  

Fig 1: Enrichment plot: KEGG\_PHOSPHATIDYLINOSITOL\_SIGNALING\_SYSTEM      
 Profile of the Running ES Score & Positions of GeneSet Members on the Rank Ordered List

  

| SYMBOL | TITLE | RANK IN GENE LIST | RANK METRIC SCORE | RUNNING ES | CORE ENRICHMENT || 1 | PRKCG | na | 203 | 0.657 | 0.0515 | No |
| 2 | PIK3R5 | na | 1058 | 0.434 | 0.0648 | No |
| 3 | CALML6 | na | 1514 | 0.391 | 0.0859 | No |
| 4 | DGKH | na | 1684 | 0.375 | 0.1138 | No |
| 5 | INPP4B | na | 2823 | 0.299 | 0.1071 | No |
| 6 | DGKB | na | 4824 | 0.223 | 0.0689 | No |
| 7 | DGKZ | na | 6049 | 0.182 | 0.0494 | No |
| 8 | SYNJ2 | na | 6226 | 0.176 | 0.0598 | No |
| 9 | PIK3CD | na | 6778 | 0.159 | 0.0578 | No |
| 10 | ITPKA | na | 6807 | 0.158 | 0.0708 | No |
| 11 | PLCB3 | na | 7444 | 0.141 | 0.0648 | No |
| 12 | PIKFYVE | na | 8727 | 0.111 | 0.0375 | No |
| 13 | PIP5K1A | na | 9172 | 0.102 | 0.0336 | No |
| 14 | DGKD | na | 9445 | 0.096 | 0.0342 | No |
| 15 | PLCD1 | na | 9845 | 0.089 | 0.0304 | No |
| 16 | INPP1 | na | 11201 | 0.067 | -0.0028 | No |
| 17 | PIK3CB | na | 11439 | 0.064 | -0.0041 | No |
| 18 | ITPR3 | na | 12500 | 0.049 | -0.0304 | No |
| 19 | PLCD3 | na | 12883 | 0.044 | -0.0376 | No |
| 20 | PLCG1 | na | 13642 | 0.035 | -0.0565 | No |
| 21 | CALM2 | na | 13884 | 0.032 | -0.0606 | No |
| 22 | PIK3CA | na | 14021 | 0.031 | -0.0619 | No |
| 23 | CALML5 | na | 15459 | 0.014 | -0.1021 | No |
| 24 | IPPK | na | 15968 | 0.009 | -0.1160 | No |
| 25 | PIK3R1 | na | 16145 | 0.007 | -0.1204 | No |
| 26 | PLCZ1 | na | 16755 | 0.001 | -0.1379 | No |
| 27 | PI4KA | na | 16869 | -0.000 | -0.1411 | No |
| 28 | PIK3C2A | na | 16932 | -0.001 | -0.1428 | No |
| 29 | INPPL1 | na | 17188 | -0.003 | -0.1499 | No |
| 30 | INPP5K | na | 17197 | -0.004 | -0.1498 | No |
| 31 | CALML3 | na | 17310 | -0.005 | -0.1526 | No |
| 32 | SYNJ1 | na | 17629 | -0.007 | -0.1612 | No |
| 33 | CALM3 | na | 18318 | -0.014 | -0.1798 | No |
| 34 | INPP5D | na | 18494 | -0.015 | -0.1835 | No |
| 35 | IMPA1 | na | 18555 | -0.016 | -0.1839 | No |
| 36 | PIP5K1C | na | 18811 | -0.018 | -0.1896 | No |
| 37 | INPP5E | na | 19291 | -0.023 | -0.2014 | No |
| 38 | PTEN | na | 19758 | -0.028 | -0.2125 | No |
| 39 | INPP4A | na | 19981 | -0.030 | -0.2163 | No |
| 40 | PIP4K2B | na | 20260 | -0.033 | -0.2214 | No |
| 41 | DGKQ | na | 20482 | -0.035 | -0.2248 | No |
| 42 | INPP5A | na | 21183 | -0.042 | -0.2413 | No |
| 43 | PLCB4 | na | 21374 | -0.044 | -0.2430 | No |
| 44 | PIP4K2C | na | 21562 | -0.046 | -0.2444 | No |
| 45 | PI4KB | na | 21741 | -0.048 | -0.2453 | No |
| 46 | ITPR2 | na | 21984 | -0.051 | -0.2479 | No |
| 47 | PIK3R2 | na | 22894 | -0.061 | -0.2688 | No |
| 48 | PIK3C3 | na | 22999 | -0.062 | -0.2664 | No |
| 49 | CDS2 | na | 23252 | -0.065 | -0.2680 | No |
| 50 | CDS1 | na | 23495 | -0.068 | -0.2691 | No |
| 51 | PIK3C2B | na | 23777 | -0.072 | -0.2709 | No |
| 52 | PRKCA | na | 23805 | -0.072 | -0.2653 | No |
| 53 | OCRL | na | 24140 | -0.077 | -0.2682 | No |
| 54 | PLCG2 | na | 24806 | -0.087 | -0.2798 | No |
| 55 | ITPR1 | na | 25459 | -0.098 | -0.2901 | No |
| 56 | INPP5B | na | 25592 | -0.100 | -0.2852 | No |
| 57 | PIP4K2A | na | 25647 | -0.101 | -0.2779 | No |
| 58 | ITPK1 | na | 26034 | -0.108 | -0.2797 | No |
| 59 | CALM1 | na | 26557 | -0.117 | -0.2845 | No |
| 60 | IMPA2 | na | 27058 | -0.127 | -0.2878 | No |
| 61 | CDIPT | na | 27382 | -0.134 | -0.2854 | No |
| 62 | DGKA | na | 27735 | -0.142 | -0.2832 | No |
| 63 | DGKG | na | 28176 | -0.153 | -0.2825 | No |
| 64 | PLCD4 | na | 28431 | -0.160 | -0.2758 | No |
| 65 | ITPKB | na | 29842 | -0.201 | -0.2989 | Yes |
| 66 | INPP5J | na | 30054 | -0.208 | -0.2868 | Yes |
| 67 | PIK3CG | na | 30335 | -0.218 | -0.2759 | Yes |
| 68 | DGKE | na | 30971 | -0.241 | -0.2732 | Yes |
| 69 | PIK3C2G | na | 31109 | -0.246 | -0.2556 | Yes |
| 70 | PLCB2 | na | 31149 | -0.248 | -0.2351 | Yes |
| 71 | PLCB1 | na | 32371 | -0.315 | -0.2428 | Yes |
| 72 | PIK3R3 | na | 32895 | -0.352 | -0.2271 | Yes |
| 73 | PRKCB | na | 34175 | -0.520 | -0.2186 | Yes |
| 74 | PLCE1 | na | 34473 | -0.637 | -0.1716 | Yes |
| 75 | DGKI | na | 34571 | -0.701 | -0.1131 | Yes |
| 76 | PIP5K1B | na | 34727 | -1.347 | 0.0000 | Yes |
Table: GSEA details [plain text format]

  

Fig 2: KEGG\_PHOSPHATIDYLINOSITOL\_SIGNALING\_SYSTEM      
 Blue-Pink O' Gram in the Space of the Analyzed GeneSet

  

Fig 3: KEGG\_PHOSPHATIDYLINOSITOL\_SIGNALING\_SYSTEM: Random ES distribution      
 Gene set null distribution of ES for **KEGG\_PHOSPHATIDYLINOSITOL\_SIGNALING\_SYSTEM**
